# Analysis of the decoupling effect and driving factors of carbon emissions from the transportation sector in Guangdong Province

**DOI:** 10.1038/s41598-023-45492-7

**Published:** 2023-10-31

**Authors:** Yongping Tang, Huiyuan Jiang

**Affiliations:** 1 Sanya Science and Education Innovation Park, Wuhan University of Technology, Sanya, 572025 Hainan China; 2 School of Transportation and Logistics Engineering, Wuhan University of Technology, Wuhan, 430063 Hubei China

**Keywords:** Climate change, Climate-change impacts, Climate-change mitigation

## Abstract

To propel the green transformation of the transportation industry in Guangdong Province. Against the backdrop of the five-year plan, this study employs the Tapio decoupling model and the Logarithmic Mean Divisia Index decomposition method to analyze the decoupling status and driving factors of carbon emissions from transportation and economic growth in Guangdong Province from 2001 to 2020. The results show that:(1) Both the total volume of carbon emissions from transportation and per capita emissions exhibit an overall upward trend, with petroleum-based emissions accounting for an overwhelming 96%, with diesel emissions register the highest proportion and most substantial increase. (2) The decoupling status predominantly manifests as weak decoupling, with intermittent years expansive coupling,, expansive negative decoupling, strong decoupling, and recessive coupling, thus indicating a persistent state of decoupling instability. (3) The study introduces the urbanization effect, wherein the effects of income urbanization, as well as spatial urbanization, predominantly exert positive driving influences. Conversely, the effects of energy intensity, population urbanization, industry scale, and energy structure collectively exert negative driving influences. Notably, the effect of income urbanization emerges as the primary factor propelling the augmentation of carbon emissions, while the energy intensity effect serves as the primary factor curbing such escalation. Finally, pertinent policy recommendations are put forth.

Global climate change has emerged as an urgent challenge to human survival and societal progress. During the 75th United Nations General Assembly in September 2020, China announced its commitment to peak carbon dioxide emissions before 2030 and strive to achieve carbon neutrality by 2060. China is currently the world's largest emitter of carbon emissions^1^, with a significantly larger reduction target compared to other countries. The transportation sector in China contributes approximately one-tenth of the country's total carbon emissions and is a fundamental, leading, strategic, and important service industry within the national economy. It is also one of the fastest-growing industries in terms of energy consumption and carbon emissions, making it the second-largest source of global carbon emissions^2,3^.

From 2000 to 2015, Guangdong Province had the highest carbon emissions from the transportation sector among all provinces in China, accounting for approximately 9.46% of the national transportation sector CO_2_ emissions^4^. With the acceleration of urbanization and the continuous growth of rigid transportation demands, the rapid development of the transportation sector imposes a significant burden on the environment. This will inevitably lead to increased energy consumption and intensified carbon emissions, hindering ecological and environmental benefits and impeding high-quality economic development. Therefore, it is imperative to explore a path of green and sustainable development. Guangdong Province not only leads the country in terms of economic development, but is also a province with a large population, resulting in increased energy consumption and carbon emissions^5,6^. Moreover, it was one of the earliest regions in China to carry out low-carbon construction pilot projects, emphasizing environmental protection and implementing a series of measures^7,8^. Achieving coordinated development between the transportation sector and energy carbon emissions in Guangdong Province is a key issue in resolving the contradiction between economic growth and environmental protection.

Therefore, analyzing the decoupling relationship between carbon emissions from the transportation sector and industry economic growth in Guangdong Province, as well as investigating the driving factors influencing decoupling and their contributions to the decoupling index, will contribute to the promotion of green transformation and sustainable development in the transportation sector of Guangdong Province. This analysis will provide a practical basis for selecting feasible paths and formulating policies for low-carbon development tailored to the transportation sector in Guangdong Province.

## Literature review

The synergistic development of the transportation sector and environmental governance has long been a focal point of research, and scholars both domestically and internationally have employed various methods to study this topic. Early studies on the relationship between transportation carbon emissions and economic growth primarily focused on issues such as coupling coordination, interactive mechanisms, and the Environmental Kuznets Curve (EKC)^9–13^. However, with the accelerated urbanization process and the rapid development of the transportation sector, scholars both domestically and internationally have undertaken research on the decoupling status between carbon emissions from the transportation sector and economic development. Compared to the EKC approach, decoupling analysis is considered more straightforward and capable of dynamically revealing the real-time relationship across different years. Consequently, it is widely regarded as a more appropriate method for assessing the relationship between carbon emissions and economic growth^14,15^. The concept of decoupling was initially introduced by the Organization for Economic Co-operation and Development (OECD) in their research on energy policy issues. It reflects the degree to which economic development and environmental pressures change in synchrony. The decoupling model enables annual quantitative analysis of the dependency of carbon emissions on economic growth and has significant advantages in terms of computation^16^. Therefore, in this study, the Tapio decoupling model is employed to analyze the decoupling relationship between carbon emissions from the transportation sector and the economic development of the industry in Guangdong Province.

In studies on the relationship between transportation carbon emissions and economic growth, Tapio^17^ examined the decoupling relationship between economic growth and carbon emissions in the European transportation sector, proposing a theoretical framework that distinguishes decoupling, negative decoupling, and coupling. It further divides them into eight types, including weak decoupling, strong decoupling, and expansive coupling. Wang et al.^18^ investigated the decoupling status of China's transportation industry and found that the decoupling status in the eastern region was more stable and ideal compared to the central and western regions. Wang et al.^19^ applied the Tapio model to study the decoupling relationship between economic growth and carbon emissions in the transportation industry of Jiangsu Province, revealing a distinct cyclical pattern of weak decoupling, expansive negative decoupling, and weak decoupling during the period from 1995 to 2013. Through in-depth analysis, these changes were found to be related to the policies implemented during the same period by the government. Hu et al.^20^ utilized the Tapio decoupling model to analyze the decoupling status between transportation carbon emissions and economic growth in the Yangtze River Economic Belt from 2004 to 2018. The results indicated that the decoupling of transportation carbon emissions in the Yangtze River Economic Belt was primarily weak decoupling and overall unstable. Yi et al.^21^ studied data from 30 provinces in China and examined the relationship between the development of the transportation industry and carbon emissions from a provincial perspective using the Tapio model. The findings suggested that underdeveloped provinces were more likely to exhibit a weak decoupling status compared to developed provinces and coastal provinces. It is evident that the overall relationship between transportation sector carbon emissions and economic growth varies among different regions, particularly with significant differences in decoupling status. In this study, we investigate the decoupling relationship between carbon emissions from the transportation sector and economic development in Guangdong Province, taking the five-year plan as the background.

In studies on the driving factors of transportation carbon emissions, commonly used research methods include the Structural Decomposition Analysis (SDA) and the Index Decomposition Analysis (IDA)^19^. The SDA method requires input-output data for decomposing influencing factors and has higher data requirements compared to the IDA method^22^. On the other hand, the IDA method offers advantages such as simpler data requirements and better data availability. Within the IDA framework, the Logarithmic Mean Divisia Index (LMDI) method^23–27^ is widely employed in studies on the changes in energy consumption and environmental pollution in the transportation sector. Wang et al.^28^ found that energy intensity played a promoting role in decoupling in developed countries in Europe. However, for developing Asian countries, energy intensity acted as a main factor inhibiting carbon emissions. Kim^29^ discovered that the increase in carbon emissions in the transportation sector in South Korea from 1990 to 2013 was primarily influenced by the effect of economic growth, while the inhibition of carbon emissions was mainly influenced by the energy intensity effect in the transportation sector. Kaya^30^ argued that population, per capita GDP, energy use per unit of GDP, and carbon emissions per unit of energy use were the four main factors driving carbon emissions in a country or region. Raza et al.^31^ employed the LMDI model to examine the influencing factors of carbon emissions in the transportation sector of Pakistan, revealing that economic growth was a significant factor promoting carbon emissions. Talbi^32^ studied the impact of fuel energy consumption, transportation energy intensity, economic growth, urbanization process, and fuel mix on carbon emissions in the transportation sector of Tunisia, with energy efficiency and fuel mix playing dominant roles in reducing carbon emissions. Jia et al.^33^ applied the LMDI method and took a multi-sectors and multi-stages approach, conducted a study on the driving factors of carbon emissions in China since 1980. The results suggest that the energy and industrial structure within the transportation sector have the capacity to mitigate the increase in carbon emissions. Yang et al.^34^ utilized the LMDI decomposition model and identified that the sustained expansion of economic scale was the primary driving factor behind carbon emissions growth in the Yangtze River Economic Belt's transportation sector, while the transformation of transportation and economic structure served as key factors inhibiting carbon emissions growth, with technological advancements contributing to the suppression of carbon emissions. Li et al.^35^ conducted research on the driving factors of carbon emissions in the Beijing-Tianjin-Hebei region's transportation sector, finding that per capita GDP had the largest driving effect on carbon emissions, while energy intensity had the most pronounced inhibitory effect. Yang et al.^36^ analyzed the influencing factors of carbon emissions in the transportation sector of Jiangsu Province using the LMDI method, revealing that economic output, population scale, and industrial structure positively drove the increase in carbon emissions, while energy structure and energy intensity negatively influenced the increase in carbon emissions. Wu et al.^37^ applied the LMDI method to conduct a multi-factor decomposition of carbon emissions changes in Shanghai's transportation sector, and the results indicated that both population size and per capita GDP played a promoting role in carbon emissions changes, while energy intensity and energy structure exerted inhibitory effects. As carbon emissions can vary due to different energy structures of the research subjects, this study selects eight energy types and introduces the concept of urbanization to explore the contribution values of driving factors and decoupling indices in transportation carbon emissions in Guangdong Province.

Through a review of previous research, we found that the current research on carbon emissions in the transportation sector has made some progress, but there are also the following three deficiencies and improvements: (1) Existing research on the driving factors of transportation carbon emissions in Guangdong Province has primarily employed the LMDI method for decomposition analysis, lacking the integration with decoupling models. The introduction of decoupling theory provides a new research perspective for analyzing the changes between economic growth and carbon emissions, as well as the calculation and analysis of the decoupling index contribution values of various driving factors. This study adopts the Tapio decoupling model and LMDI index decomposition method, replacing traditional econometric models. Compared to traditional measurement methods, the calculations of the Tapio decoupling model and LMDI method are more concise and can dynamically and instantaneously reflect the annual contributions of various influencing factors to carbon emission changes, providing more intuitive results. (2) Previous research has mainly focused on analyzing the driving factors of carbon emissions in terms of energy structure, energy intensity, industry scale, economic growth, and population change, neglecting the impact of urbanization on the transportation sector. Urbanization in Guangdong Province is rapidly progressing, and considering the rapid expansion of urban space, resident income, population density, and industrial agglomeration, the rapid advancement of the urbanization process has a significant influence on carbon emissions in the transportation sector. Therefore, analyzing the impact of urbanization contributes to a more comprehensive understanding of carbon reduction and decoupling goals in the transportation industry in Guangdong Province. (3) This study takes a comparative analysis approach during the Five-Year Plan period to explore the differences in decoupling status between carbon emissions in the transportation sector and industry economic development in Guangdong Province. Based on this analysis, we delve into the reasons behind these differences during the Fifteen-Year Plan to the Thirteenth Five-Year Plan period, providing references for future studies on transportation carbon emissions in various provinces.

This study aims to provide a comprehensive understanding of the decoupling relationship between carbon emissions from the transportation sector and industry economic development in Guangdong Province by utilizing the Tapio decoupling model. Based on the calculation of carbon emissions from the transportation sector in Guangdong Province from 2001 to 2020, the study improves upon the traditional LMDI decomposition model by incorporating the concept of elastic decoupling. The decoupling index is decomposed into six driving factors, with a focus on examining the impacts of urbanization, energy structure, and energy intensity on the decoupling status of the transportation sector in Guangdong Province. By combining the Tapio decoupling model with the LMDI factor decomposition method, a decoupling factor decomposition model is constructed to analyze the decoupling indexes of each factor and quantify their contributions. Finally, policy recommendations and strategies are proposed, providing a scientific basis and assessment reference for the formulation and implementation of policies suitable for the low-carbon development of transportation in Guangdong Province.

## Methods

### Accounting method

Due to the inherent mobility of carbon dioxide emissions, the calculation of carbon dioxide emissions in the transportation sector involves more uncertainties compared to other industries^38^. According to the National Greenhouse Gas Inventory Guidelines by the Intergovernmental Panel on Climate Change (IPCC)^39^, two methods, namely the top-down and bottom-up approaches, are provided for estimating carbon dioxide emissions in the transportation sector. The top-down method involves multiplying the transportation energy consumption within a specific region by the carbon emission factors of various energy sources to obtain the total carbon emissions. On the other hand, the bottom-up method calculates the total energy consumption by multiplying the distance traveled by different modes of transportation with the unit energy consumption per distance, and then multiplying it by the carbon emission factors of various energy sources to obtain the total carbon emissions^37^. Due to the limited availability of data on vehicle stocks, travel distances, and unit fuel consumption per distance in the bottom-up method, this study adopts the top-down approach to estimate the carbon emissions from energy consumption in the transportation sector of Guangdong province:

1 $$C = \sum\limits_{i = 1}^{8} {e_{i} \times k_{i} } = \sum\limits_{i = 1}^{8} {e_{i} } \times v_{i} \times f_{i} \times r_{i} \times \frac{44}{{12}}$$

Here, $C$ represents the ${\text{CO}}_{2}$ emissions of the transportation sector in Guangdong Province, $i$ represents the type of energy consumption, $e_{i}$ represents the energy consumption in the transportation sector, $k_{i}$ represents the carbon emission factor, $v_{i}$ represents the average lower heating value, $f_{i}$ represents the carbon content per unit heat value, $r_{i}$ represents the carbon conversion rate, and $44/12$ represents the ratio of the molecular weight of carbon dioxide to carbon.

This study takes a more comprehensive approach than previous research^40,41^ in considering the carbon emissions of the transportation sector in Guangdong Province. The calculation process takes into account eight types of energy sources: raw coal, gasoline, kerosene, diesel oil,, fuel oil, liquefied petroleum gas, natural gas, and electricity. The carbon emission factor for electricity is derived from the average CO_2_ emission factor of regional power grids in China^42^. The relevant parameters involved in the calculation of carbon emission factors are presented in Supplementary Table S1.

### Decoupling model

The decoupling theory proposed by the OECD aims to depict the disconnection between economic growth and the environment, wherein economic growth no longer entails an increase in resource consumption or environmental pollution^43^. This article investigates the relationship between carbon emissions in the transportation sector of Guangdong Province and economic development, using the Tapio decoupling model^17^. The decoupling expression is formulated as follows:

2 $$t\left( {C,TGDP} \right) = \frac{\% \Delta C}{{\% \Delta TGDP}} = \frac{{(C^{t} { - }C^{0} )/C^{0} }}{{(TGDP^{t} {\text{ - T}}GDP^{0} )/TGDP^{0} }}$$

In the equation, $t\left( {C,TGDP} \right)$ denotes the decoupling elasticity index between carbon emissions and the value added in the transportation sector. ${\text{\% }}\Delta C$ and ${\text{\% }}\Delta TGDP$ represent the respective rates of change for carbon emissions and value added in the transportation sector. $C^{t}$ and $TGDP^{t}$ denote the carbon emissions and value added in the corresponding period, while $C^{0}$ and $TGDP^{0}$ represent the carbon emissions and value added in the base period of the study. Based on the values of the elasticity index, the decoupling status can be classified into eight categories, as illustrated in Supplementary Figure S1.

### LMDI model of decoupled state drive factor

The Kaya identity is an analytical method proposed by Japanese scholar Yoichi Kaya^30^ to explore the factors influencing carbon dioxide emissions, expressing carbon dioxide emissions in mathematical relationships with energy, economy, and population. The urbanization rate of Guangdong Province has risen from 55.00% in 2000 to 74.15% in 2020. The rapid development of urbanization has had a significant impact on transportation carbon emissions. Building upon previous research findings^20,36,37,40,41,44^, this study incorporates the concept of urbanization. The changes in transportation carbon emissions caused by economic growth and lifestyle changes during the urbanization process are referred to as income urbanization effects, while the changes in transportation carbon emissions resulting from population density are termed population urbanization effects. Furthermore, the changes in transportation carbon emissions due to spatial expansion are referred to as spatial urbanization effects. Considering the characteristics of carbon emissions in Guangdong Province's transportation sector, the Kaya identity is expanded, decomposing the factors influencing transportation carbon emissions into six elements: energy structure, energy intensity, industry scale, income urbanization effects, population urbanization effects, and spatial urbanization effects. The improved LMDI model is constructed as follows:

3 $$\begin{gathered} C^{t} = \sum\limits_{i = 1}^{8} {\frac{{C_{i}^{t} }}{{E_{i}^{t} }}} \times \frac{{E_{i}^{t} }}{{E^{t} }} \times \frac{{E^{t} }}{{TGDP^{t} }} \times \frac{{TGDP^{t} }}{{GDP^{t} }} \times \frac{{GDP^{t} }}{{P^{t} }} \times \frac{{P^{t} }}{{AREA^{t} }} \times AREA^{t} \\ = \sum\limits_{i = 1}^{8} {CF_{i}^{t} } \times ES_{i}^{t} \times EI^{t} \times TU^{t} \times GP^{t} \times PR^{t} \times R^{t} \\ \end{gathered}$$

where, $C_{i}^{t}$ and $E_{i}^{t}$ represents the carbon emissions and energy consumption of the i type of energy in the transportation sector in the $t$ th year.$E^{t}$ represents the total energy consumption in the transportation sector in the $t$ th year.$TGDP^{t}$ represents the added value of the transportation sector in the $t$ th year.$GDP^{t}$ represents the regional gross domestic product in the $t$ th year.$P^{t}$ represents the urban resident population in the $t$ th year.$AREA^{t}$ represents the built-up area of the city in the $t$ th year. Among these variables, $CF_{i}^{t}$ represents the carbon emission coefficient for the i type of energy, which is a constant, resulting in a contribution of 0 to the changes in transportation-related carbon emissions.$ES_{i}^{t}$ represents the proportion of energy consumption for the i type of energy in the total energy consumption, reflecting the energy structure effect.$EI^{t}$ represents the energy intensity of the transportation industry per unit of gross domestic product, representing the energy intensity effect. $TU^{t}$ represents the share of the transportation industry's added value in the total output, signifying the industry scale effect.$GP^{t}$ represents per capita GDP, representing the income urbanization effect.$PR^{t}$ represents the population density in the built-up area, indicating the population urbanization effect.$R^{t}$ represents the impact of spatial expansion on carbon emissions, representing the spatial urbanization effect.

By employing the residue-free LMDI method proposed by Ang B.W.^23–27^, we can perform a decomposition of Eq. (3) to express the changes in transportation carbon emissions relative to the base period. The resulting expression is as follows:

4 $$\Delta C = C^{t} - C^{0} = \Delta ES + \Delta EI + \Delta TU + \Delta GP + \Delta PR + \Delta R$$

By applying logarithmic transformations, addition/subtraction operations, and further decomposition to Eq. (4), we can obtain the contributions of various factors to carbon emissions in the transportation sector. The expressions for the contributions of these decomposition factors are as follows:

5 $$\Delta ES = \sum\limits_{i = 1}^{8} {\frac{{C_{i}^{t} - C_{i}^{0} }}{{\ln C_{i}^{t} - \ln C_{i}^{0} }}} \ln \frac{{ES_{i}^{t} }}{{ES_{i}^{0} }}$$

6 $$\Delta EI = \sum\limits_{i = 1}^{8} {\frac{{C_{i}^{t} - C_{i}^{0} }}{{\ln C_{i}^{t} - \ln C_{i}^{0} }}} \ln \frac{{EI_{{}}^{t} }}{{EI_{{}}^{0} }}$$

7 $$\Delta TU = \sum\limits_{i = 1}^{8} {\frac{{C_{i}^{t} - C_{i}^{0} }}{{\ln C_{i}^{t} - \ln C_{i}^{0} }}} \ln \frac{{TU^{t} }}{{TU^{0} }}$$

8 $$\Delta GP = \sum\limits_{i = 1}^{8} {\frac{{C_{i}^{t} - C_{i}^{0} }}{{\ln C_{i}^{t} - \ln C_{i}^{0} }}} \ln \frac{{GP^{t} }}{{GP^{0} }}$$

9 $$\Delta PR = \sum\limits_{i = 1}^{8} {\frac{{C_{i}^{t} - C_{i}^{0} }}{{\ln C_{i}^{t} - \ln C_{i}^{0} }}} \ln \frac{{PR^{t} }}{{PR^{0} }}$$

10 $$\Delta R = \sum\limits_{i = 1}^{8} {\frac{{C_{i}^{t} - C_{i}^{0} }}{{\ln C_{i}^{t} - \ln C_{i}^{0} }}} \ln \frac{{R^{t} }}{{R^{0} }}$$

In Eq. (4), $\Delta C$ represents the change in transportation carbon emissions, while $C_{i}^{t}$ and $C_{i}^{0}$ represent the carbon emissions in periods t and the base period, respectively. If the contributions of the various factor effects are greater than 0, it indicates that the corresponding factor will cause an increase in transportation carbon emissions. Conversely, if the contributions are negative, it implies that the factor will suppress transportation carbon emissions.

In order to further understand the contributions of various driving factors to the decoupling elasticity index between carbon emissions in the transportation sector and industry economic development, we can substitute Eq. (4) into Eq. (2) to derive an expanded decoupling model for carbon emissions in the transportation sector and industry economic growth. The derived equation is as follows:

11 $$\begin{aligned} T\left( {c,tgdp} \right) & = \frac{{\frac{\Delta C}{{C^{0} }}}}{{\frac{\Delta TGDP}{{TGDP^{0} }}}} = \frac{{\frac{{C^{t} { - }C^{0} }}{{C^{0} }}}}{{\frac{{TGDP^{t} {\text{ - T}}GDP^{0} }}{{TGDP^{0} }}}} \\ & = \frac{{\frac{\Delta ES}{{C^{0} }}}}{{\frac{\Delta TGDP}{{TGDP^{0} }}}} + \frac{{\frac{\Delta EI}{{C^{0} }}}}{{\frac{\Delta TGDP}{{TGDP^{0} }}}} + \frac{{\frac{\Delta TU}{{C^{0} }}}}{{\frac{\Delta TGDP}{{TGDP^{0} }}}} + \frac{{\frac{\Delta GP}{{C^{0} }}}}{{\frac{\Delta TGDP}{{TGDP^{0} }}}} + \frac{{\frac{\Delta PR}{{C^{0} }}}}{{\frac{\Delta TGDP}{{TGDP^{0} }}}} + \frac{{\frac{\Delta R}{{C^{0} }}}}{{\frac{\Delta TGDP}{{TGDP^{0} }}}} \\ & = T_{es}^{t} + T_{ei}^{t} + T_{tu}^{t} + T_{gp}^{t} + T_{pr}^{t} + T_{r}^{t} \\ \end{aligned}$$

where, $T_{es}^{t}$ represents the contribution of the energy structure effect to the decoupling elasticity index, indicating the influence of structural adjustments on the decoupling relationship. $T_{ei}^{t}$ represents the contribution of the energy intensity effect to the decoupling elasticity index, reflecting the impact of technological progress on the decoupling relationship. $T_{tu}^{t}$ represents the contribution of the industry scale effect to the decoupling elasticity index, indicating the influence of industry development on the decoupling relationship. $T_{gp}^{t}$, $T_{pr}^{t}$ and $T_{r}^{t}$ represent the contributions of the income urbanization effect, population urbanization effect, and spatial urbanization effect, respectively, to the decoupling elasticity index. These contributions are used to examine the impact of urbanization development on the decoupling index. If the contributions of these effects are greater than 0, it implies that the respective factor hinders the decoupling process. Conversely, if the contributions are negative, it indicates that the factor promotes decoupling, driving the decoupling state towards the desired direction.

### Data sources

The main focus of this study is to investigate the decoupling relationship and driving factors between carbon emissions in the transportation sector and industry economic development in Guangdong Province. The research covers the period from 2001 to 2020. The energy consumption in the transportation sector includes energy consumption in transportation, warehousing, and postal services. The data on transportation energy consumption are obtained from the China Energy Statistical Yearbook for the years 2002 to 2021. Economic data are sourced from the China Statistical Yearbook, the Guangdong Statistical Yearbook for the years 2002 to 2021. The data on the built-up area of cities are obtained from the China Urban Construction Statistical Yearbook for the years 2001 to 2020. Additionally, to eliminate the impact of price fluctuations on GDP, this study adjusts the actual GDP and the total output of the transportation industry based on the corresponding production indices using the year 2001 as the base year. The selection of carbon emission coefficients is crucial for accurately calculating carbon emissions. To reflect the actual situation in Guangdong Province more accurately, this study adopts carbon emission coefficients from the Provincial Greenhouse Gas Inventory and Energy Statistical Yearbook instead of using data from the IPCC. The average low heating value is sourced from the General Rules for Comprehensive Energy Calculation, while the carbon content and carbon oxidation rate per unit of heat are obtained from the Guidelines for Provincial Greenhouse Gas Inventory Compilation.

## Result analysis

### Changes in carbon emissions

**Figure 1:**
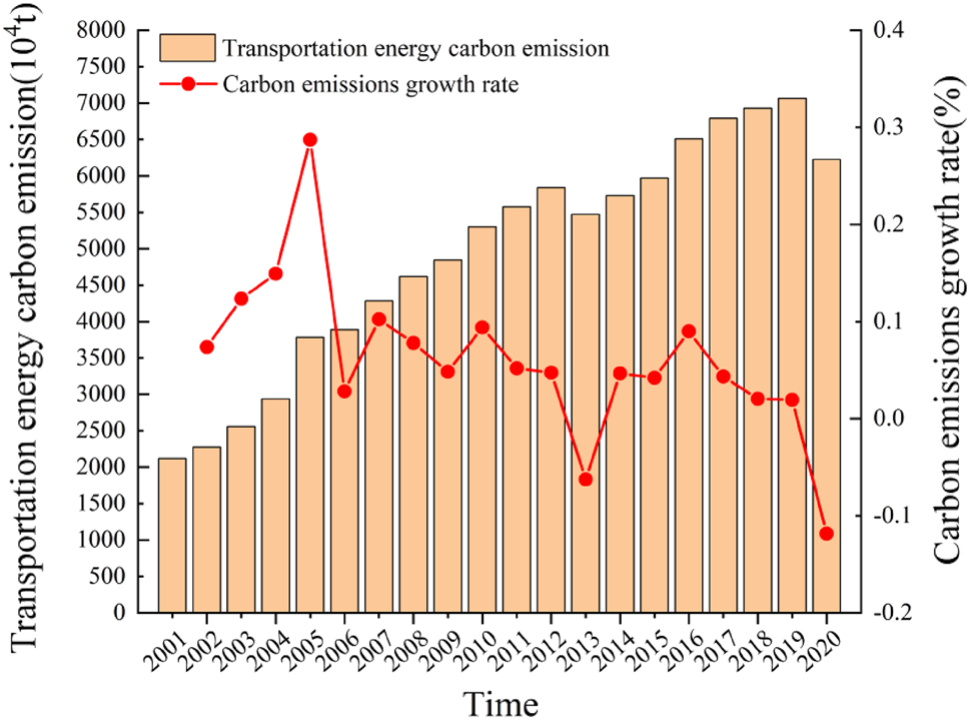
Carbon emissions and growth rate of transportation in Guangdong Province from 2001 to 2020.

Based on the China Energy Statistical Yearbook and the Guangdong Statistical Yearbook, we have calculated the relevant data for carbon emissions in the transportation sector in Guangdong Province from 2001 to 2020. As shown in Table 1 and Fig. 1, the total carbon emissions and per capita carbon emissions in the transportation sector in Guangdong Province exhibited an overall upward trend during the period. The total carbon emissions increased from 2117.97 thousand tons to 6226.40 thousand tons, with an average annual growth rate of 5.84%, while per capita carbon emissions rose from 0.24 kg to 0.49 kg, with an average annual growth rate of 3.81%.The higher carbon emissions compared to the study by Zhuang et al.^40^ are due to the fact that this paper includes raw coal, liquefied petroleum gas, natural gas, and electricity. Additionally, it analyzes the contribution of each energy source to carbon emissions in the transportation sector. This comprehensive consideration of energy sources aligns more closely with reality, providing a more accurate assessment of carbon emissions.

**Table 1:** Carbon emissions from transportation in Guangdong province from 2001 to 2020.

Time	Total carbon emissions	Per capita carbon emissions	Raw coal	Gasoline	Kerosene	Diesel oil	Fuel Oil	Electricity
2001	2117.97	0.24	13.82	588.33	272.70	955.43	193.08	12.68
2002	2274.58	0.26	2.58	644.19	295.45	1046.20	190.07	15.34
2003	2555.81	0.29	1.69	682.57	340.99	1228.64	192.89	27.17
2004	2937.74	0.32	2.47	779.51	369.87	1452.29	211.03	33.42
2005	3781.68	0.41	2.66	1091.30	431.56	1905.19	235.28	16.77
2006	3888.17	0.41	2.85	1172.06	431.56	1905.19	252.69	17.89
2007	4286.02	0.44	3.17	1301.58	479.24	2083.73	280.62	20.52
2008	4619.74	0.47	3.38	1392.67	512.80	2263.81	300.25	21.76
2009	4843.41	0.48	3.55	1459.51	537.43	2372.48	314.67	24.90
2010	5299.06	0.51	3.88	1589.73	585.38	2584.18	374.47	29.97
2011	5574.16	0.52	4.10	1682.61	619.57	2735.17	362.77	35.97
2012	5838.63	0.53	4.62	1749.91	697.01	2803.52	408.11	37.53
2013	5473.14	0.49	5.02	966.75	757.64	3047.42	516.16	39.70
2014	5729.13	0.50	8.19	1063.42	782.42	3147.08	533.02	41.61
2015	5970.14	0.51	8.40	1178.05	803.15	3230.48	547.16	45.41
2016	6507.21	0.55	8.63	1455.00	859.35	3436.51	714.66	27.88
2017	6789.73	0.56	8.86	1499.79	882.13	3439.27	733.46	60.31
2018	6927.97	0.56	9.05	1530.62	901.96	3498.09	749.95	68.82
2019	7062.50	0.57	8.48	1438.30	924.84	3701.46	723.10	81.66
2020	6226.40	0.49	7.75	1287.28	824.94	3272.09	652.24	80.95

Due to the incomplete data of some transportation energy sources in the energy balance table of Guangdong region in the China Energy Statistics Yearbook 2002–2021, the carbon emissions from energy consumption such as liquefied petroleum gas and natural gas have been calculated in this paper and are not included in the above table.

Specifically, during the period from 2001 to 2010, the total carbon emissions in the transportation sector in Guangdong Province increased by 3181.10 thousand tons, showing a rapid upward trend, and reaching the highest growth rate within the study period in 2005. From 2011 to 2020, the growth rate of carbon emissions did not exceed 9%, with negative growth observed during the periods of 2012–2013 and 2019–2020. The year 2020 marked the lowest growth rate of carbon emissions within the study period. Analyzing the changes in carbon emissions in the transportation sector in Guangdong Province during the study period, several factors may have contributed to these changes. Firstly, during the Tenth Five-Year Plan and Eleventh Five-Year Plan periods, the rapid urbanization and economic growth accelerated, leading to a continuous increase in the rigid demand for transportation, which in turn expanded the demand for energy consumption and contributed to the rise in carbon emissions from Guangdong's transportation sector. Secondly, government authorities have been increasingly focused on energy-saving and emission reduction in the transportation sector, as evidenced by the inclusion of binding targets in development plans during the Twelfth Five-Year Plan and Thirteenth Five-Year Plan periods. Various policies and regulations were introduced to facilitate energy reform and promote industrial restructuring, which, to some extent, mitigated the growth rate of carbon emissions from energy consumption in Guangdong's transportation sector. However, there still exists a significant contradiction between overall economic development and carbon reduction efforts.

**Figure 2:**
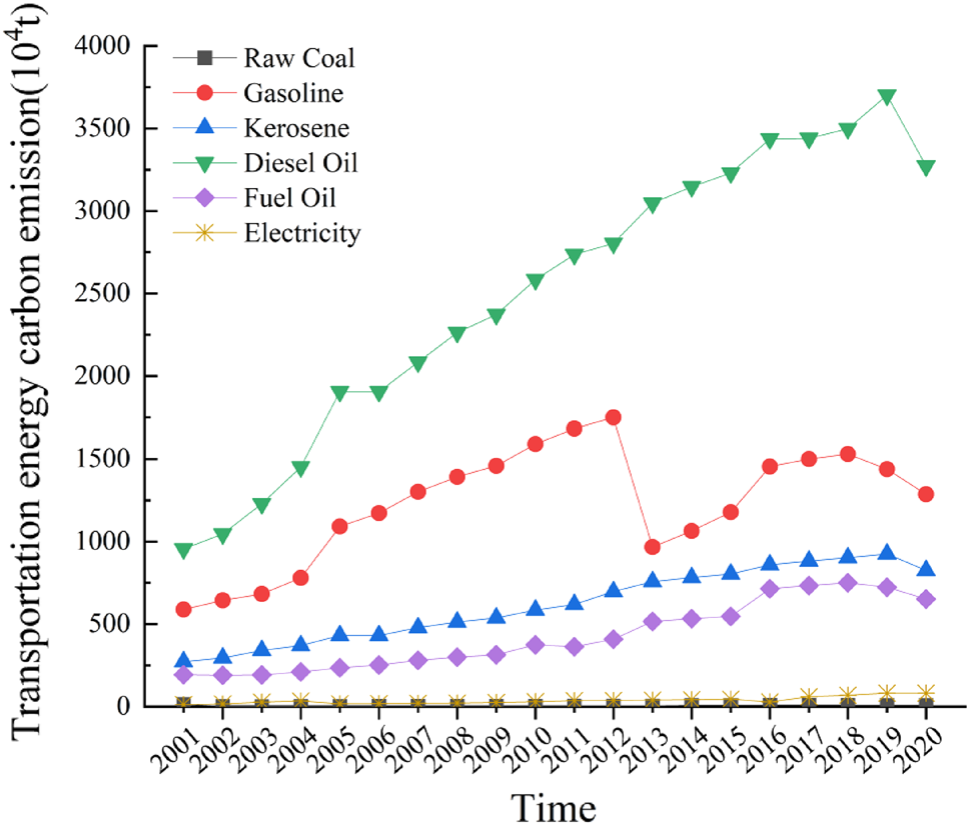
Carbon emission trends of various types of transportation energy consumption in Guangdong Province from 2001 to 2020.

According to Fig. 2, it can be observed that diesel fuel experienced the largest increment and growth rate among various types of transportation energy consumption in Guangdong Province from 2001 to 2020. In 2013, the proportion of diesel oil consumption accounted for a significant 55.68% of the total carbon emissions from transportation. This can be attributed to the increasing demand for large-scale truck freight, maritime transport, and intercity transportation. On the other hand, gasoline consumption initially showed a slow rising trend but experienced a rapid decline in 2013. This can be attributed to the rapid economic development during the Tenth Five-Year Plan and the Eleventh Five-Year Plan, which led to an increase in vehicle purchases and subsequently resulted in a rapid growth of carbon emissions. However, during the Twelfth Five-Year Plan, the implementation of the Guangdong Province Implementation Plan for Total Emission Reduction of Vehicle Pollutants during the Twelfth Five-Year Plan strictly controlled the additional carbon emissions from motor vehicles and implemented measures to enhance vehicle management and emission reduction, effectively mitigating carbon emissions from motor vehicles.The carbon emissions from kerosene and fuel oil consumption showed a slow but steady increasing trend, primarily due to the expansion of aviation transportation using kerosene as fuel and the expansion of waterway transportation using fuel oil, such as the expansion of Guangzhou Baiyun International Airport, Shenzhen Bao'an International Airport, and the ports of Guangzhou and Shenzhen. The contribution of raw coal consumption to carbon emissions in the transportation sector averaged less than 1% of the total carbon emissions. The consumption of liquefied petroleum gas, natural gas, and electricity in transportation accounted for less than 3% of the total carbon emissions on average. However, petroleum-based energy consumption accounted for a substantial 96% of the total carbon emissions in transportation. Therefore, it can be observed that petroleum-based energy consumption has the greatest contribution to carbon emissions in the transportation sector in Guangdong Province, and to a certain extent, it determines the changing trends of carbon emissions in the transportation sector.

### Decoupling analysis

The decoupling situation between carbon emissions in the transportation sector and industry economic development in Guangdong Province from 2001 to 2020 was calculated using Eq. (2). The results of the calculations are presented in Table 2.

**Table 2:** Decoupling state of carbon emissions and economic development of transportation sector in Guangdong Province from 2001 to 2020.

Time	$\Delta C/C$	$\Delta TGDP/TGDP$	$t\left( {C,TGDP} \right)$	Decoupling states
2001–2002	0.07	0.08	0.92	Expansive coupling
2002–2003	0.12	0.06	2.21	Expansive negative decoupling
2003–2004	0.15	0.13	1.15	Expansive coupling
2004–2005	0.29	0.19	1.53	Expansive negative decoupling
2005–2006	0.03	0.16	0.17	Weak decoupling
2006–2007	0.10	0.11	0.92	Expansive coupling
2007–2008	0.08	0.08	0.96	Expansive coupling
2008–2009	0.05	0.05	0.99	Expansive coupling
2009–2010	0.09	0.11	0.83	Expansive coupling
2010–2011	0.05	0.12	0.44	Weak decoupling
2011–2012	0.05	0.13	0.38	Weak decoupling
2012–2013	− 0.06	0.07	− 0.83	Strong decoupling
2013–2014	0.05	0.11	0.43	Weak decoupling
2014–2015	0.04	0.06	0.69	Weak decoupling
2015–2016	0.09	0.08	1.10	Expansive coupling
2016–2017	0.04	0.09	0.49	Weak decoupling
2017–2018	0.02	0.06	0.32	Weak decoupling
2018–2019	0.02	0.07	0.29	Weak decoupling
2019–2020	− 0.12	− 0.03	3.48	Recessive coupling

From Table 2, we can observe that during the study period, the decoupling status between carbon emissions in the transportation sector and industry economic development in Guangdong Province can be classified into five categories: expansive coupling, expansive negative decoupling, weak decoupling, strong decoupling, and recessive coupling. The decoupling index shows a fluctuating trajectory resembling an M curve. Among these decoupling statuses, weak decoupling is the predominant category, indicating that the growth rate of carbon emissions in the transportation sector has been slower than the economic growth rate of the transportation industry over a considerable period. Overall, we can identify three distinct phases:

1. From 2001 to 2010, the first phase exhibited three decoupling statuses: expansive coupling, expansive negative decoupling, and weak decoupling, with expansive coupling being the predominant one. The reason for this can be attributed to the emphasis on improving the efficiency of economic growth during the Tenth Five-Year Plan and the Eleventh Five-Year Plan, which led to rapid expansion of industries characterized by heavy industrialization in Guangdong Province. This expansion resulted in an increase in inflexible transportation and logistics demands, accompanied by inadequate transportation infrastructure and a lack of effective emission reduction measures. Consequently, transportation energy consumption was difficult to control, exacerbating carbon emissions from the transportation sector.
2. From 2010 to 2015, the second phase was primarily characterized by weak decoupling, with a strong decoupling status observed in 2012 to 2013, representing an improvement compared to the first phase. The reasons for this improvement can be attributed to the national focus on energy conservation and emission reduction during this period. Guangdong Province began to prioritize low-carbon development and introduced policies and regulations such as the Guangdong Province Development Plan for Energy Conservation and Emission Reduction in the Transportation Sector during the Twelfth Five-Year Plan and the Guangdong Province Implementation Plan for Total Emission Reduction of Vehicle Pollutants during the Twelfth Five-Year Plan. These measures aimed to promote the application of new energy vehicles in the public transport sector, the development and utilization of clean and renewable energy, optimization of freight transportation organization, adjustment of transportation structure, and control of pollution from ocean-going vessels. Additionally, the continuous optimization of industrial structure in Guangdong Province led to a gradual decline in energy consumption per unit of output. These initiatives partially restrained the increase in carbon emissions from transportation, effectively mitigated the growth rate of carbon emissions, and improved the decoupling status between carbon emissions from transportation and industry economic development.
3. From 2015 to 2020, the third phase exhibited three decoupling statuses: expansive coupling, weak decoupling, and recessive coupling, indicating an unstable decoupling status of carbon emissions from transportation. The reasons for this can be attributed to the sustained economic development in Guangdong Province along with intensified environmental governance efforts. Measures such as energy transformation in transportation, acceleration of energy-saving and emission reduction technology innovation, and enhancement of regulatory and service capabilities for energy-saving and emission reduction were implemented as part of the Guangdong Province Development Plan for Energy Conservation and Emission Reduction in the Transportation Sector during the Thirteenth Five-Year Plan. However, during this period, Guangdong Province also faced challenges including a sharp increase in the number of motor vehicles, increased maritime traffic, rapid growth in passenger and freight volume, exponential growth in tourism and logistics transportation, all of which exerted significant pressure on carbon emission reduction in the transportation sector. It is evident that more efforts are needed to achieve substantial reductions in transportation emissions in Guangdong Province. It is worth noting that a recession decoupling status was observed in 2019-2020, mainly due to the economic decline in the transportation sector and reduced passenger and freight volume caused by the impact of the COVID-19 pandemic at the end of 2019.

Based on the analysis above, it is evident that the year 2010 marked a turning point, indicating the decisive role of five-year plan policies in carbon emissions reduction. However, the overall economic development of the transportation industry in Guangdong Province still remains dependent on carbon emissions from transportation. Striking a balance between industry economic growth and reducing transportation carbon emissions has been a persistent challenge in the development of the transportation sector in Guangdong Province. In the future, it will be crucial to continue optimizing industrial and energy structures, improving energy efficiency, and adhering to a path of low-carbon transformation and development.

### Analysis of the drivers of decoupling relationship

**Figure 3:**
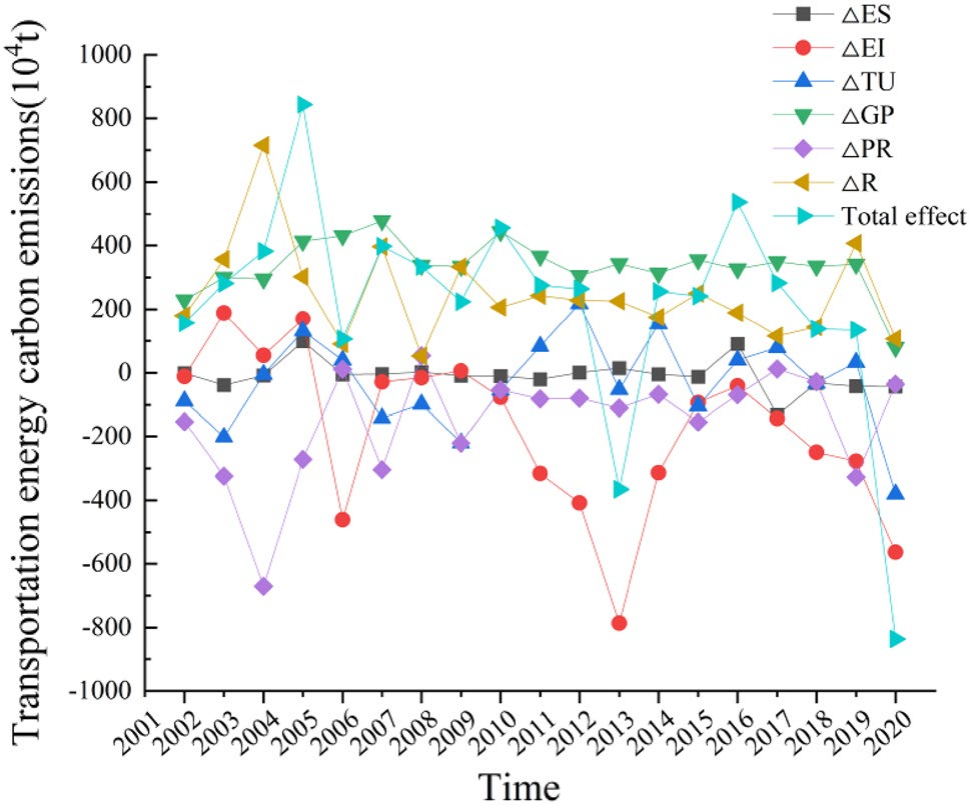
Contribution value of each driver of transportation carbon emissions in Guangdong from 2001 to 2020.

**Figure 4:**
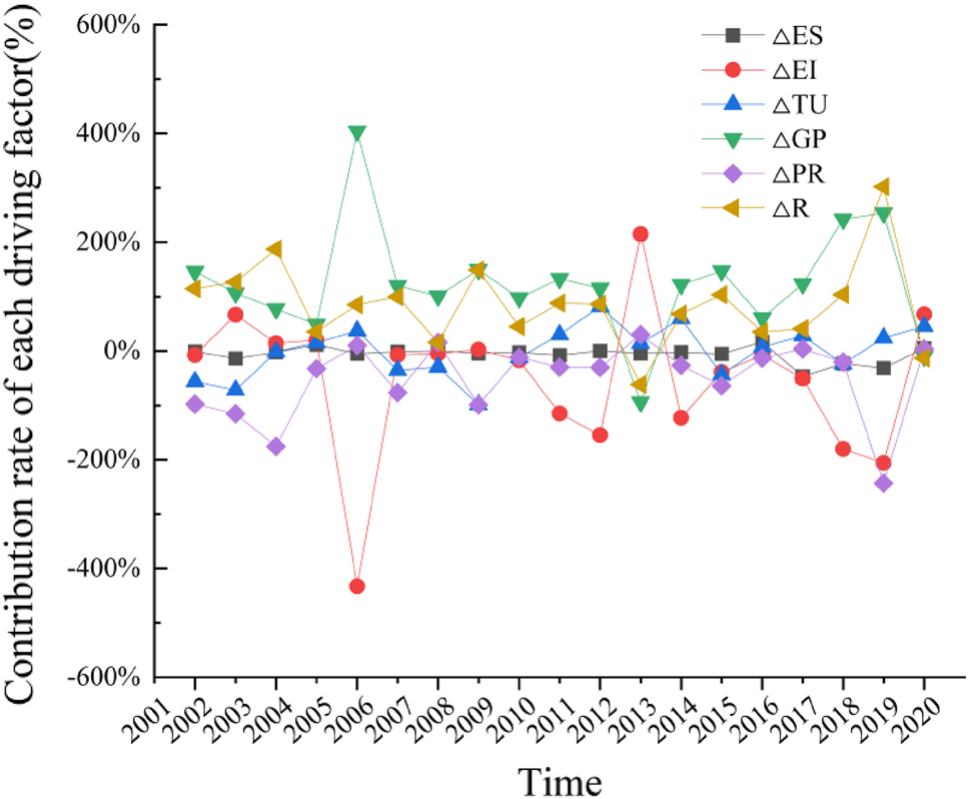
Contribution rate of each driver of transportation carbon emissions in Guangdong from 2001 to 2020.

The decoupling elasticity index is limited to studying the synchronous relationship between carbon emissions in the transportation sector and industry economic development, but it cannot explain the mechanism of carbon emission changes. Current research on carbon emissions in Guangdong Province's transportation sector has not considered the impact of urbanization effects. Zhuang et al.^40^ and Deng et al.^41^ considered factors such as energy structure, energy intensity, industry scale, economic growth, and population size in their research. In contrast, this paper introduces the concept of urbanization, improves the LMDI model, and obtains the contributions and contribution rates of six driving factors: energy structure, energy intensity, industry scale, income urbanization effect, population urbanization effect, and spatial urbanization effect on carbon emissions in the transportation sector of Guangdong Province, as shown in Table 3, Figs. 3, and 4.

**Table 3:** Decomposition of carbon emission drivers in Guangdong transportation from 2001 to 2020.

Time	$\Delta ES$		$\Delta EI$		$\Delta TU$		$\Delta GP$		$\Delta PR$		$\Delta R$		Total effect
	Value	Ratio	Value	Ratio	Value	Ratio	Value	Ratio	Value	Ratio	Value	Ratio	
2002	− 1.11	− 0.71%	− 11.09	− 7.08%	− 87.59	− 55.93%	229.22	146.36%	− 152.73	− 97.52%	179.91	114.88%	156.61
2003	− 38.36	− 13.64%	188.19	66.92%	− 201.43	− 71.63%	300.16	106.73%	− 324.11	− 115.25%	356.78	126.86%	281.23
2004	− 8.33	− 2.18%	55.14	14.44%	− 4.85	− 1.27%	295.08	77.26%	− 670.50	− 175.55%	715.40	187.31%	381.94
2005	98.93	11.72%	169.84	20.12%	131.85	15.62%	412.91	48.93%	− 271.55	− 32.18%	301.95	35.78%	843.93
2006	− 5.09	− 4.78%	− 460.81	− 432.72%	39.84	37.41%	430.47	404.23%	11.35	10.66%	90.73	85.20%	106.49
2007	− 3.74	− 0.94%	− 28.28	− 7.11%	− 140.90	− 35.41%	477.78	120.09%	− 303.94	− 76.40%	396.93	99.77%	397.85
2008	2.25	0.68%	− 15.19	− 4.55%	− 97.73	− 29.29%	337.87	101.24%	53.80	16.12%	52.72	15.80%	333.72
2009	− 8.92	− 3.99%	6.28	2.81%	− 220.28	− 98.48%	334.73	149.65%	− 221.22	− 98.90%	333.07	148.91%	223.67
2010	− 10.26	− 2.25%	− 76.61	− 16.81%	− 54.34	− 11.93%	443.75	97.39%	− 52.92	− 11.61%	206.03	45.22%	455.65
2011	− 20.36	− 7.40%	− 315.65	− 114.74%	83.21	30.25%	366.32	133.16%	− 81.46	− 29.61%	243.04	88.35%	275.10
2012	0.72	0.27%	− 408.18	− 154.34%	217.06	82.07%	305.68	115.58%	− 79.11	− 29.91%	228.30	86.32%	264.47
2013	14.66	− 4.01%	− 786.09	215.08%	− 51.97	14.22%	342.69	− 93.76%	− 109.87	30.06%	225.10	− 61.59%	− 365.49
2014	− 4.79	− 1.87%	− 313.50	− 122.46%	153.71	60.04%	312.81	122.19%	− − 67.09	− 26.21%	174.86	68.31%	256.00
2015	− 12.42	− 5.15%	− 92.87	− 38.53%	− 103.80	− 43.07%	354.67	147.16%	− 153.92	− 63.86%	249.34	103.46%	241.00
2016	90.64	16.88%	− 40.72	− 7.58%	40.12	7.47%	326.47	60.79%	− 68.47	− 12.75%	189.03	35.20%	537.07
2017	− 131.42	− 46.52%	− 142.41	− 50.41%	79.29	28.07%	349.24	123.62%	11.95	4.23%	115.88	41.02%	282.52
2018	− 31.47	− 22.76%	− 249.31	− 180.35%	− 32.18	− 23.28%	335.26	242.52%	− 27.81	− 20.12%	143.76	103.99%	138.24
2019	− 41.86	− 31.11%	− 277.12	− 205.98%	32.85	24.42%	341.26	253.66%	− 327.22	− 243.22%	406.62	302.24%	134.53
2020	− 42.88	5.13%	− 563.78	67.43%	− 380.27	45.48%	79.51	− 9.51%	− 35.43	4.24%	106.74	− 12.77%	− 836.10
total	− 153.79	− 3.74%	− 3362.16	− 81.84%	− 597.44	− 14.54%	6375.88	155.19%	− 2870.24	− 69.86%	4716.19	114.79%	4108.44

According to Table 3 and Figs. 3 and 4, there are significant variations in the impact of different driving factors on the total change in carbon emissions from transportation in Guangdong Province from 2001 to 2020. The urbanization effect has a much greater influence on carbon emissions than other factors. When ranked in descending order of impact, the contributions are as follows: income urbanization effect with a contribution rate of 155.19%; spatial urbanization effect with a contribution rate of 114.79%; energy intensity effect with a contribution rate of -81.84%; population urbanization effect with a contribution rate of -69.86%; industry scale effect with a contribution rate of -14.54%; and energy structure effect with a contribution rate of -3.74%.

Analyzing the trends of these decomposition effects in relation to the overall effect, it can be observed that the income urbanization effect and spatial urbanization effect are consistently positive, exerting a significant positive driving force on changes in transportation carbon emissions. The cumulative annual effects of these factors are 6375.88 thousand tons and 4716.19 thousand tons, respectively. This indicates that as the residents' living standards improve, there is a substantial increase in transportation demand and energy consumption. The continuous growth in demand for housing mobility and the expansion of urban construction contribute to the increase in transportation carbon emissions. On the other hand, the energy intensity effect, population urbanization effect, industry scale effect, and energy structure effect mostly have negative values. These factors collectively contribute to the negative driving force on changes in transportation carbon emissions, with cumulative annual effects of -3362.16 thousand tons, -2870.24 thousand tons, -597.44 thousand tons, and -153.79 thousand tons, respectively. This indicates that reducing the energy consumption per unit of GDP in the transportation sector, controlling the population density in urban areas, decreasing the proportion of the transportation industry in regional GDP, and optimizing the proportion of different energy sources in total transportation energy consumption will restrain the increase in transportation carbon emissions.

**Figure 5:**
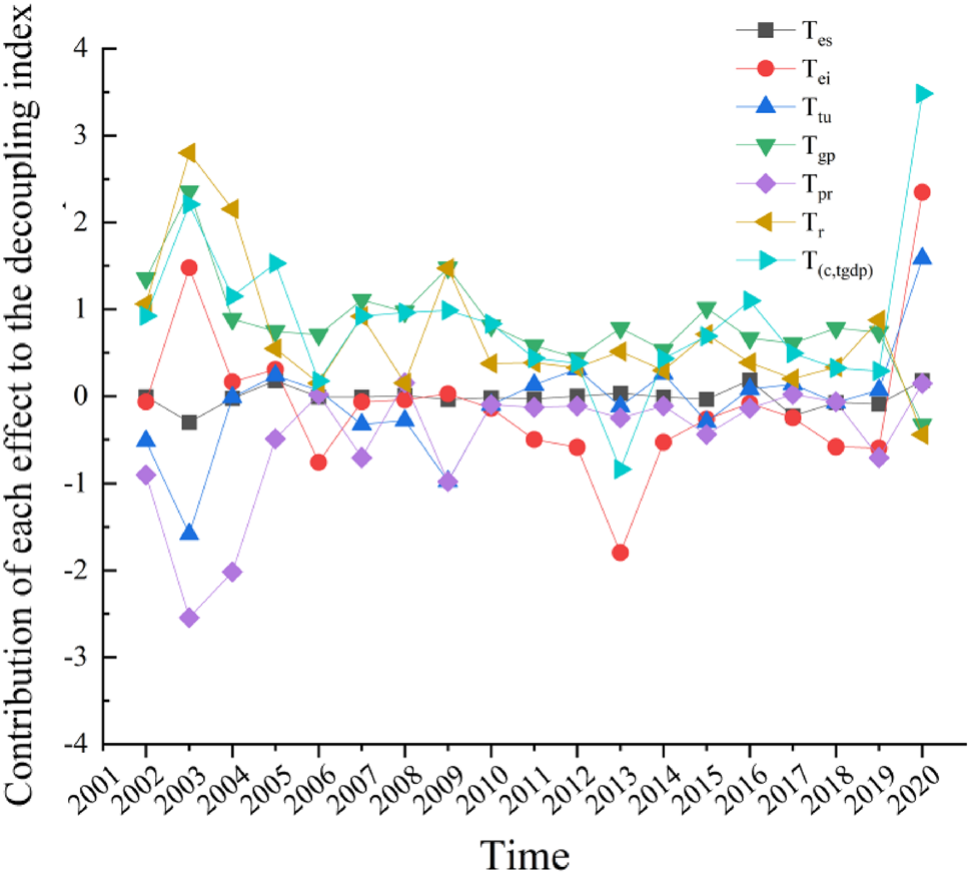
Trend of the total decoupling index and the contribution value of each effect to the decoupling index.

In comparison to previous studies, Zhuang et al.^40^ and Deng et al.^41^ solely employed the LMDI method to analyze the driving factors behind carbon emissions in Guangdong Province's transportation sector. They lacked integration with the decoupling model. In this study, we have effectively combined the Tapio model with the LMDI method to provide a more intuitive exploration of the contribution rates of various driving factors to changes in carbon emissions in Guangdong Province's transportation sector. In accordance with Eq. (11), the decoupling indices for various effects mentioned above are further derived. These decoupling indices represent the contribution and changing trends of each driving factor to the decoupling elasticity between transportation carbon emissions and economic development in Guangdong Province from 2001 to 2020. The results can be found in Table 4 and Fig. 5. Overall, the decoupling indices for the income urbanization effect and spatial urbanization effect are predominantly positive. Particularly noteworthy is the high decoupling index of 2.356 for the income urbanization effect in 2002 to 2003, which hinders the decoupling of carbon emissions from transportation and industry economic growth. On the other hand, the decoupling indices for the energy intensity effect, population urbanization effect, industry scale effect, and energy structure effect are mostly negative, promoting the decoupling of carbon emissions from transportation and industry economic growth. These findings contribute to the progression of decoupling toward a decoupled state.

**Table 4:** Decoupling index of various drivers of transportation carbon emissions in Guangdong from 2001 to 2020.

Time	$T_{es}$	$T_{ei}$	$T_{tu}$	$T_{gp}$	$T_{pr}$	$T_{r}$	$T_{{\left( {c,tgdp} \right)}}$
2001–2002	− 0.007	− 0.065	− 0.517	1.353	− 0.901	1.062	0.924
2002–2003	− 0.301	1.477	− 1.581	2.356	− 2.544	2.801	2.208
2003–2004	− 0.025	0.166	− 0.015	0.888	− 2.018	2.153	1.150
2004–2005	0.179	0.308	0.239	0.748	− 0.492	0.547	1.528
2005–2006	− 0.008	− 0.757	0.065	0.707	0.019	0.149	0.175
2006–2007	− 0.009	− 0.066	− 0.326	1.107	− 0.704	0.920	0.922
2007–2008	0.006	− 0.044	− 0.282	0.973	0.155	0.152	0.961
2008–2009	− 0.039	0.028	− 0.973	1.479	− 0.977	1.471	0.988
2009–2010	− 0.019	− 0.140	− 0.099	0.811	− 0.097	0.376	0.833
2010–2011	− 0.032	− 0.501	0.132	0.581	− 0.129	0.385	0.436
2011–2012	0.001	− 0.586	0.312	0.439	− 0.114	0.328	0.380
2012–2013	0.033	− 1.795	− 0.119	0.783	− 0.251	0.514	− 0.835
2013–2014	− 0.008	− 0.530	0.260	0.529	− 0.114	0.296	0.433
2014–2015	− 0.036	− 0.266	− 0.297	1.015	− 0.440	0.713	0.690
2015–2016	0.185	− 0.083	0.082	0.667	− 0.140	0.386	1.097
2016–2017	− 0.230	− 0.249	0.138	0.610	0.021	0.202	0.493
2017–2018	− 0.074	− 0.583	− 0.075	0.784	− 0.065	0.336	0.323
2018–2019	− 0.090	− 0.597	0.071	0.735	− 0.705	0.876	0.290
2019–2020	0.179	2.348	1.584	− 0.331	0.148	− 0.445	3.482

## Discussion

### Energy structure effect analysis

The energy structure effect plays a negative driving role and is one of the factors promoting decoupling. During the study period, the contribution of the energy structure effect to carbon emissions from transportation in Guangdong Province alternated between positive and negative values, resulting in a cumulative negative contribution. This indicates that reducing the proportion of various energy consumption in total transportation energy consumption can restrain the increase in carbon emissions from transportation and promote the decoupling of carbon emissions from transportation and industry economic growth. However, the cumulative contribution rate is only − 3.74%, indicating a very weak effect. Based on Table 1 and Fig. 2, it can be observed that high-carbon energy consumption dominated from 2001 to 2020, while the consumption of clean energy remained relatively low. Looking at the trends over the years, diesel oil consumption consistently accounted for the largest proportion, with an average exceeding 50%, particularly in 2013, when diesel oil energy consumption accounted for a staggering 56% of total carbon emissions from transportation. Additionally, the proportion of clean energy consumption has increased slightly but with limited impact. The share of electricity consumption rose from 0.6% in 2001 to 1.3% in 2020. Due to the continued reliance on fossil fuels such as diesel oil and gasoline for energy consumption in the transportation sector, the energy structure remains relatively monolithic. Therefore, the task of optimizing the energy consumption structure in Guangdong Province's transportation sector remains challenging. Currently, there is an urgent need to promote the low-carbon development of the transportation industry in Guangdong Province, intensify efforts to increase the proportion of renewable energy and clean energy in transportation, and reduce dependence on fossil fuels.

### Energy intensity effect analysis

The negative impact of energy intensity effect plays a crucial role in promoting decoupling. The energy intensity effect decreased from -11.09 thousand tons to -563.78 thousand tons, resulting in a substantial reduction of 552.68 thousand tons. Examining Table 5, we can observe that although the energy consumption in the transportation sector of Guangdong Province increased from 1057.23 thousand tons of standard coal to 3395.70 thousand tons of standard coal between 2001 and 2020, indicating a gradual upward trend, there was a notable decline in the energy consumption per unit of GDP in the transportation sector of Guangdong Province from 1.61 tons of standard coal per ten thousand yuan to 1.01 tons of standard coal per ten thousand yuan. This overall downward trend demonstrates a significant improvement in the efficiency of energy utilization in the transportation sector, which serves as a crucial pathway to achieving the decoupling of transportation carbon emissions from industry's economic growth.

**Table 5:** Terminal energy consumption and transportation industry in Guangdong province and energy consumption per unit of GDP from 2001 to 2020.

Time	TGDP	Total end-use energy consumption in the transportation sector	Energy consumption per unit of GDP in the transportation sector	GDP	Total end-use energy consumption	Energy consumption per unit of GDP
2001	657.08	1057.23	1.61	12,126.59	10,178.87	0.84
2002	709.65	1151.88	1.62	13,630.29	11,354.70	0.83
2003	749.39	1324.28	1.77	15,647.57	13,099.29	0.84
2004	846.81	1555.90	1.84	17,713.05	15,210.47	0.86
2005	1006.01	1922.78	1.91	20,228.30	17,769.37	0.88
2006	1167.97	1986.56	1.70	23,242.32	19,765.22	0.85
2007	1297.62	2191.04	1.69	26,728.67	21,912.11	0.82
2008	1402.73	2372.42	1.69	29,535.18	23,071.83	0.78
2009	1471.46	2546.35	1.73	32,459.16	24,653.69	0.76
2010	1637.73	2796.90	1.71	36,516.55	27,195.14	0.74
2011	1832.62	2916.18	1.59	40,241.24	28,479.99	0.71
2012	2061.70	2950.94	1.43	43,581.27	29,144.01	0.67
2013	2216.33	3385.94	1.53	47,285.67	30,179.70	0.64
2014	2455.69	3034.23	1.24	50,973.96	29,593.26	0.58
2015	2605.49	3152.46	1.21	55,051.87	30,145.49	0.55
2016	2819.14	3510.58	1.25	59,180.76	31,240.75	0.53
2017	3067.23	3607.82	1.18	63,619.32	32,341.66	0.51
2018	3260.46	3708.77	1.14	67,945.43	33,330.30	0.49
2019	3478.91	3814.82	1.10	72,158.05	34,141.89	0.47
2020	3360.63	3395.70	1.01	73,817.69	34,502.92	0.47

Further analysis of Fig. 3 reveals that the contribution of energy intensity effect to carbon emissions from transportation in Guangdong Province exhibits both positive and negative trends, displaying distinct phases. From 2001 to 2005, the contribution value shifted from negative to positive, likely attributed to the significant energy consumption resulting from the rapid economic development during the Tenth Five-Year Plan period. From 2005 to 2010, the contribution value alternated between positive and negative, possibly due to limitations in the technological advancement of the transportation sector at that time. From 2010 to 2020, the contribution value consistently remained negative, with an overall increasing trend in its impact on promoting carbon reduction in transportation. Moreover, the gap between the inhibiting effect on carbon emissions and the driving effect of income urbanization has gradually diminished. When combined with the information from Fig. 1, it becomes evident that although the carbon emissions from transportation in Guangdong Province have continued to increase during this period, the growth rate has significantly slowed. This indicates that the province has made strides in improving the efficiency of energy utilization in transportation and transitioning towards a low-energy, high-efficiency operational model, leading to a mitigating effect on carbon emissions. These achievements reflect the significant outcomes in realizing the objectives of energy intensity reduction and carbon emission intensity reduction outlined in policies and regulations such as the Twelfth Five-Year Plan for Energy Conservation and Emission Reduction in Transportation in Guangdong Province and the Thirteenth Five-Year Plan for Energy Conservation and Emission Reduction in Transportation in Guangdong Province.

Energy intensity is a key indicator for measuring energy efficiency. From Table 5, it is evident that the energy consumption per unit of GDP in the transportation industry in Guangdong Province is significantly higher than the energy consumption per unit of GDP in Guangdong Province as a whole. This indicates that in the short term, energy efficiency remains a prominent issue constraining the development of low-carbon transportation in Guangdong Province. Efforts need to be intensified in improving energy utilization efficiency in the transportation sector. Furthermore, Fig. 3 reveals a strong correlation between the contribution of the energy intensity effect to the decoupling index and the trends in decoupling transportation carbon emissions. This further emphasizes that the decoupling status of transportation carbon emissions is primarily influenced by the energy intensity effect. In the future, Guangdong Province should focus on reducing the energy intensity in the transportation sector, continuously enhancing emission reduction technologies, improving energy utilization efficiency in transportation, and promoting the decoupling of transportation carbon emissions from industry economic growth.

### Industry scale effect analysis

The industry scale effect has a negative driving effect and is one of the factors that promote decoupling. During the study period, the industry scale effect on carbon emissions in the transportation sector of Guangdong Province contributed both positively and negatively, with a cumulative negative contribution. This indicates that the industry scale effect has a restraining effect on the growth of carbon emissions in the transportation sector of Guangdong Province. However, the cumulative contribution rate is relatively low at -14.54%, suggesting a limited impact compared to other driving factors. From Table 3, it can be observed that the cumulative carbon emission effect due to the scale factor from 2001 to 2020 is approximately -597.44 thousand tons, with an annual average carbon emission reduction effect of about -31.44 thousand tons. With the implementation of relevant policies from the Twelfth Five-Year Plan to the Thirteenth Five-Year Plan, Guangdong Province has optimized its industrial layout, strictly controlled energy-intensive and high-emission industries, and strengthened the management of key industries and areas such as transportation and public institutions for energy conservation and emission reduction. Clean production has been extensively promoted in key sectors and companies, with the widespread application of clean production technologies. It can be inferred that over the past two decades, Guangdong Province has adjusted the scale of the transportation industry to reduce carbon emissions and promote the decoupling of carbon emissions from industry economic growth. Additionally, from Table 5, despite the increase in value added in the transportation industry in Guangdong Province from 657.08 hundred million in 2001 to 3360.63 hundred million in 2020, a growth of 5.11 times, the proportion of the transportation industry's output value to the regional GDP decreased from 5.42% to 4.55% during the period 2001 to 2020, a reduction of 0.87%. This indicates that over the past two decades, the proportion of the transportation industry in Guangdong Province has gradually declined. As an energy-intensive industry, the decrease in its proportion in GDP has facilitated decoupling. However, the scale of the industry is a long-term dynamic process and has lagging effects. It would be quite challenging to achieve significant reductions in transportation carbon emissions and promote decoupling by drastically adjusting the scale of the transportation industry. Moreover, in the impact of income urbanization on carbon emissions, some of the effects may be attributed to the scale factor of the transportation industry, which might to some extent mask the contribution of the industry scale effect to carbon emissions in transportation. Therefore, the driving effect of changes in the scale of the industry on transportation carbon emissions in Guangdong Province is not apparent.

### Analysis of income urbanization effect

The income urbanization effect plays a positive driving role and is the primary factor hindering decoupling. During the study period, the cumulative contribution of the income urbanization effect was as high as 6375.88 thousand tons, accounting for 155.19% of the total. This indicates that the increase in per capita GDP has led to an increase in carbon emissions from transportation, making the income urbanization effect the dominant factor driving the growth of carbon emissions in Guangdong Province's transportation sector.The contribution of income urbanization effects to the decoupling index aligns closely with the trend of the decoupling index for total transportation carbon emissions. This further underscores that the decoupling status of transportation carbon emissions is primarily influenced by income urbanization effects. During the research period, the per capita GDP of Guangdong Province has experienced steady growth. Correspondingly, transportation carbon emissions increased from 2117.97 thousand tons to 6226.40 thousand tons, an increase of 2.94 times. The improvement in economic development has to some extent raised the living standards of residents, thereby stimulating an increase in consumption demands for enjoyment, such as online shopping and tourism, as well as a rapid growth in private vehicles. With the expansion of transportation demand, it has not only driven the construction of transportation infrastructure and service facilities but also led to continuous growth in transportation energy consumption, further intensifying the pressure to reduce carbon emissions from transportation. Additionally, from Table 3 and Fig. 3, it can be observed that from 2010 to 2020, the contribution of the income urbanization effect to transportation carbon emissions and the decoupling index showed a fluctuating downward trend. This may be attributed to Guangdong Province's economy entering a period of transitioning growth rates and gradually shifting towards a high-quality development model that emphasizes the quality, efficiency, and sustainability of economic growth. Guangdong Province has begun to prioritize the development of a low-carbon economy and engage in industrial restructuring and optimization. In the future, the inhibiting effect of the income urbanization effect on decoupling is likely to further weaken.

### Analysis of population urbanization effect

The population urbanization effect plays a negative driving role and is one of the factors promoting decoupling. During the study period, the population urbanization effect contributed to a total of -2870.24 thousand tons and had a contribution rate of -69.86%. On one hand, the expansion rate of urban areas in Guangdong Province exceeded the population growth rate. The development of urbanization has led to the concentration of urban populations, which helps optimize urban transportation infrastructure layout, accelerate the development of public transportation, and improve transportation efficiency. On the other hand, the population urbanization effect has led to industrial agglomeration, which helps optimize logistics network layout, leverage economies of scale, promote the development of freight and logistics enterprises, and facilitate the low-carbonization of transportation equipment. This has a positive effect on reducing transportation carbon emissions. Furthermore, with the gradual relaxation of China's family planning policy and the accelerated process of population urbanization in Guangdong Province, the scope of urban construction areas continues to expand, leading to continuous population growth and increased transportation volume. Consequently, per capita energy consumption and transportation carbon emissions are bound to increase. Therefore, it is important to pay attention to the ecological and environmental impacts and pressures caused by the population urbanization effect on the increase in carbon emissions from the transportation sector. To promote decoupling between transportation carbon emissions and industry economic growth in Guangdong Province, it is necessary to reasonably control population growth to restrain the growth of transportation carbon emissions.

### Analysis of spatial urbanization effect

The spatial urbanization effect plays a positive driving role and is one of the factors hindering decoupling. The spatial urbanization effect significantly hampers the decoupling process between transportation carbon emissions and industry economic growth, contributing to a total of 4716.19 thousand tons and accounting for 114.79% of the total contributions, second only to the income urbanization effect. This phenomenon is primarily caused by the continuous expansion of urban space in Guangdong Province as urbanization progresses. This expansion increases the transportation distance between production and consumption locations, leading to a phenomenon of work-residence separation. As a result, the demand for private cars rises, increasing travel distances and adding complexity to the transportation network, thus stimulating a significant increase in transportation demand. Additionally, excessive land development, resource depletion, and lagging infrastructure construction and service facilities in the outskirts of cities contribute to increased energy consumption and carbon emissions in transportation, exerting pressure on carbon reduction efforts and hindering the decoupling of transportation carbon emissions from industry economic growth in Guangdong Province.

## Conclusions

Combining the Tapio decoupling model and the LMDI factor decomposition method, this paper analyzes the decoupling relationship between transportation carbon emissions and economic development in Guangdong Province from 2001 to 2020 as well as the drivers of decoupling, and the main conclusions are as follows:

1. From 2001 to 2020, both the total transportation carbon emissions and per capita carbon emissions in Guangdong Province showed an overall increasing trend. The growth rate of transportation carbon emissions exhibited a rapid increase from 2001 to 2005, but has been slowing down since 2006. Among various types of transportation energy consumption, carbon emissions from petroleum-based energy consumption contribute the most, accounting for 96% of the total. In particular, diesel oil fuel has shown the largest increase in both quantity and proportion, which is not conducive to accelerating the transition towards green and low-carbon development in the transportation sector of Guangdong Province
2. The decoupling status between transportation carbon emissions and industry economic development in Guangdong Province from 2001 to 2020 was generally unstable and can be divided into three stages. In the first stage, from 2000 to 2010, the decoupling elasticity index was generally high, showing three states: expansive coupling, expansive negative decoupling, and weak decoupling. The dominant state was expansive coupling, indicating an unsatisfactory decoupling status. In the second stage, from 2010 to 2015, the decoupling index decreased, indicating an improving decoupling status. The main state was weak decoupling, with a strong decoupling state achieved in 2012-2013.In the third stage, from 2015 to 2020, the decoupling index showed a fluctuating upward trend, presenting three states: expansive coupling, weak decoupling, and recessive coupling. The decoupling status remained unstable. During the study period, the decoupling index followed a M curve trajectory, indicating that the overall industry-economic development in the transportation sector of Guangdong Province has not yet overcome its reliance on transportation energy carbon emissions.
3. Overall, urbanization effect has a more significant hindering impact on the decoupling relationship in Guangdong Province compared to non-urbanization effects. Energy intensity effect, population urbanization effect, industry scale effect, and energy structure effect contribute to the decoupling of carbon emissions from economic growth in the transportation sector of Guangdong Province. However, income urbanization effect and spatial urbanization effect act as barriers to the decoupling of carbon emissions from economic growth. During the study period, energy intensity effect played the most crucial role in driving decoupling as a negative factor. Income urbanization effect played a significant role in obstructing decoupling as a positive factor. Energy structure effect made a limited contribution to carbon emissions reduction in the transportation sector. Industry scale effect had a promoting effect on carbon emissions reduction but its impact was less noticeable compared to other driving factors. Population urbanization effect had a minor promoting effect on carbon emissions reduction with a potential for further enhancement in the future. Spatial urbanization effect had a positive driving effect and is expected to remain a hindering factor for carbon emissions reduction in the transportation sector for a considerable period of time

### Policy recommendations

Based on the above findings of this paper, the following policy recommendations are proposed to promote the decoupling of economic development and carbon emissions in the transportation industry in Guangdong Province:

1. Optimize the industrial development model and strongly support the growth of a low-carbon economy. With low-carbon transformation as the goal, strict adherence to energy-saving and environmental standards is crucial. Continuous efforts should be made to optimize and upgrade the structure of traditional industries and accelerate the reform of high-energy-consuming and highly-polluting sectors, promoting the transition of industries towards energy conservation. Tilted investments, fiscal subsidies, and other measures should be taken to improve the infrastructure for new energy sources, while increasing investment in the funding and talent development of energy-saving and environmental protection technologies. Special attention should be given to the cultivation and attraction of professionals in the field of low-carbon transportation and logistics. Furthermore, the deep integration of next-generation internet technologies with traditional industries should be promoted, facilitating the digital transformation of industries and driving the transition of traditional low-end industries to medium and high-end sectors. Efforts should be made to achieve a coordinated and mutually beneficial progress in carbon emissions reduction and economic and social development. It is important to highlight that the hindering effect of urbanization on the decoupling of economic development and carbon emissions in the transportation sector of Guangdong Province is significantly stronger than non-urbanization effects. Therefore, in the process of advancing urbanization, it is essential to plan urban spatial structures rationally and strengthen overall urban road planning
2. Optimize the energy structure of transportation and establish an efficient transportation system. Control and reduce the consumption of petroleum-based energy by means such as price signaling, lowering the proportion of diesel oil and gasoline in transportation energy consumption. Expand the market for clean energy consumption, such as natural gas, electricity, and solar energy. Accelerate the research and development of new energy vehicles and clean energy vehicles, and promote their use through pilot projects, subsidies, and other measures. Tap into the emission reduction potential of energy structure effect. Improve low-carbon transportation standards and regulatory systems, encourage the development of multimodal transportation, and continue to promote the shift from road to rail and water transport for bulk goods transportation. This will facilitate the low-carbon development of multimodal transportation and comprehensive transportation systems, alleviate the pressure on traditional transportation networks, and improve transportation efficiency to reduce energy consumption and carbon emissions
3. Focus on technological innovation to enhance energy intensity effects. Under the context of innovation-driven development, actively leverage the opportunities brought by intelligent and digital technologies to promote the transformation of energy towards intelligence, conservation, and low carbon. Accelerate the construction of energy big data platforms, using digital, intelligent, and green smart grids as the foundation. Apply energy big data extensively in production and daily life to achieve rational allocation of energy resources and significantly improve energy efficiency, thereby reducing energy losses and achieving energy conservation and emission reduction goals. Increase investment in energy-saving and emission reduction technology innovation, using scientific and technological innovation and technological progress to promote the improvement of energy utilization efficiency and unlock the emission reduction potential of energy intensity effects. Strengthen international cooperation on carbon emission reduction technologies, making technological innovation a strong engine for carbon reduction. Introduce advanced clean energy technologies from abroad to achieve diversified energy supply and reduce reliance on traditional high-carbon energy sources. Technological progress can continuously enhance the energy-saving potential in energy consumption, effectively reducing end-use energy demand
4. Promote the concept of green transportation and advocate a low-carbon lifestyle. Expand green transportation awareness campaigns to guide the public in choosing low-carbon modes of transportation such as public transit, new energy vehicles, and shared bicycles, while reducing unnecessary transportation demand. Utilize advanced communication and networking technologies to reduce travel distances and frequencies, making transportation more environmentally friendly and travel more low-carbon. Encourage public participation in environmental governance, actively supervise and report high-emission and high-pollution issues, expand reporting channels, and increase penalties, achieving a low-carbon and environmentally friendly society through collective efforts. Make full use of market mechanisms such as carbon emissions trading and carbon taxes, increase vehicle purchase taxes and fuel surcharges, and promote the shift of transportation demand from energy-intensive and polluting road and air transport to low-carbon and environmentally friendly railway, waterway, and urban public transportation modes.

### Supplementary Information

Supplementary Information.

## Data Availability

The datasets used and analyzed during the current study are available from the corresponding author on reasonable request.
