# Supplementary material for: Analysis of the decoupling effect and driving factors of carbon emissions from the transportation sector in Guangdong Province
Source: Sci Rep. 2023 Oct 31;13:18744. doi: 10.1038/s41598-023-45492-7 (PMC10618480; doi:10.1038/s41598-023-45492-7)
Supplement: Supplementary file 1 — Supplementary Information. [file 41598_2023_45492_MOESM1_ESM.docx]

Appendix

| **Energy type** | **$v_{i}\left(kJ/kg\right)$** | **$f_{i}\left(TC/TJ\right)$** | **$r_{i}\left(\text{％}\right)$** | **$k_{i}\left(kg/kg\right)$** |
| --- | --- | --- | --- | --- |
| Raw coal | 20908 kJ/kg | 26.37 | 0.94 | 1.9003 kg/kg |
| Gasoline | 43070 kJ/kg | 18.9 | 0.98 | 2.9251 kg/kg |
| Kerosene | 43070 kJ/kg | 19.5 | 0.98 | 3.0179 kg/kg |
| Diesel oil | 42652 kJ/kg | 20.2 | 0.98 | 3.0959 kg/kg |
| Fuel Oil | 41816 kJ/kg | 21.1 | 0.98 | 3.1705 kg/kg |
| Liquefied Petroleum Gas | 50179 kJ/kg | 17.2 | 0.98 | 3.1013 kg/kg |
| Natural gas | 38931 kJ/m^3^ | 15.3 | 0.99 | 2.1622 kg/m^3^ |

**Supplementary Table S1.** Different energy type carbon emission coefficient calculation related parameters.


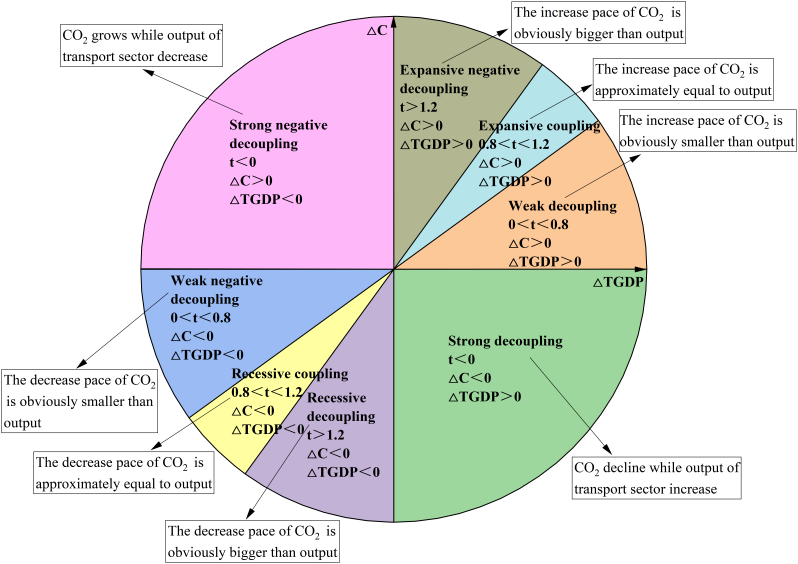


**Supplementary Figure S1.** Evaluation criteria for decoupling index.
